# Super-Reaction Enhancement of Polariton-Mediated Electron Transfer

**DOI:** 10.1021/acs.jpclett.6c03034

**Published:** 2026-10-27

**Authors:** Eric R. Koessler, Todd D. Krauss, Pengfei Huo

**Affiliations:** † Department of Chemistry, University of Rochester, Rochester, New York 14627, United States; ‡ The Institute of Optics, Hajim School of Engineering, 6927 University of Rochester, Rochester, New York 14627, United States; ¶ Center for Coherence and Quantum Science, 6927 University of Rochester, Rochester, New York 14627, United States

## Abstract

We investigate the enhancement of polariton-mediated electron transfer (PMET) using a general super-reaction mechanism where multiple donor sites locally couple to individual acceptors. We demonstrate that this enhancement extends to the collective light–matter coupling regime with a large number of acceptors and cavity-coupled donors. We use analytic rate theory and quantum dynamics simulations to show that the local super-reaction mechanism serves as an additional enhancement to the delocalized driving force enhancement of PMET. We further incorporate bath-induced disorder in the donors to investigate precisely how collective light–matter coupling protects the coherent super-reaction enhancement against disorder. The results show that ET from bright polariton states, which are separated enough from the dark state manifold, is enhanced by the super-reaction mechanism in any ET regime, offering another avenue to realize cavity modification of photochemistry.

## Graphical abstract

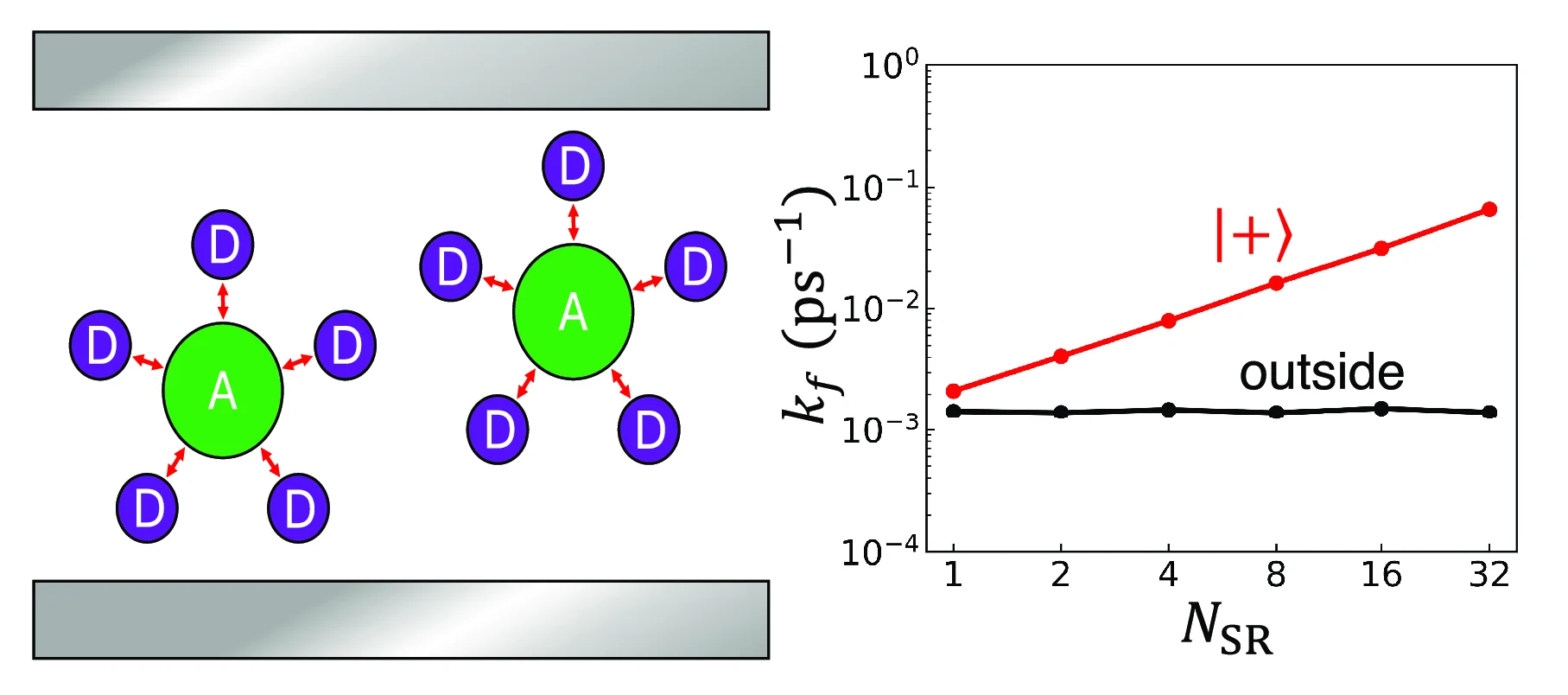

The emerging field of polariton chemistry has explored how strong coupling to optical cavities affects chemical systems.^1–9^ Recent experiments in vibrational strong coupling^10–18^ and electronic strong coupling^19–24^ have demonstrated modifications and enhancements of various chemical reactions and photophysics. Of particular ongoing interest is to experimentally demonstrate and theoretically explain chemical reaction modification in the collective strong coupling regime, where many optically active molecules are coupled to the cavity mode. This collective regime typically allows for larger light–matter coupling strengths to be attained in experiments (as opposed to few or single molecule cavity designs) but creates challenges relating to energetic disorder between molecules and enabling local chemical modifications using nonlocal cavity coupling.^24–29^

In spite of these challenges, recent work on the strong coupling of cavities to photoinduced energy and charge transfer processes has demonstrated promise in collective modifications of these transfer processes. From the theoretical side, multiple models based on the Marcus theory of electron transfer (ET) have demonstrated how collective light–matter coupling impacts the ET rate constant,^29–34^ extending beyond the single molecule strong coupling limit.^35–39^ Experimentally, there are also exciting investigations in this area, including collective modifications of photoconductivity,^40^ effective driving force,^41,42^ and energy transfer rates.^43^

In this work, we focus on investigating the possibility of super-reaction (SR) enhancement scaling and quantitatively demonstrate that the cavity can protect the enhancement from energetic disorder in the collective coupling regime. Our work builds upon ref 33 that described the concept of a super-reaction in the context of multiple donors coupled to a single acceptor inside the cavity. The super-reaction mechanism is analogous to the mechanisms of super-radiance and supertransfer in molecular aggregates,^44–47^ where multiple coupling pathways constructively interfere to enhance the effective coupling of a bright state to an acceptor state. This mechanism is distinct from other collective light–matter coupling mechanisms inside optical cavities that result in collectively modified energies for the polariton eigenstates.^1,6^ The super-reaction mechanism predicts an enhancement of the ET rate constant by a factor of *N*_D_, where *N*_D_ is the number of the donor molecules coupled to the single acceptor molecule. A super-reaction is difficult to realize outside the cavity because molecular bath fluctuations can destroy the coherent bright state that is needed to realize the super-reaction. It was qualitatively shown^33^ that the strong coupling of the donors to the cavity mode can protect the super-reaction mechanism against bath-induced disorder. We quantitatively investigate how the super-reaction mechanism and disorder affect the polariton-mediated ET (PMET) rate constants between large numbers of acceptors and cavity-coupled donors. We consider that there are *N*_A_ donor–acceptor molecular clusters instead of a single cluster. Further, we consider that the cavity mode couples collectively to a total of *N*_D_ donor molecules among these clusters through the transition dipole of the donor (such as CdSe nanoplatelets), which has been accomplished in recent experimental work.^48–51^ The light–matter interactions, as opposed to the electronic coupling, are highly nonlocal between the cavity mode and *N*_D_ donor molecules. Using a mixed quantum-classical approach,^30^ we simulate the population dynamics of the cavity-coupled super-reaction system for up to thousands of coupled donor states, which allows for insight into the large-*N* limit of the model, which is closer to the reality of most polariton chemistry experiments than few-molecule simulations. We show that super-reaction systems with many molecular clusters can experience the super-reaction enhancement inside cavities in the presence of disorder, provided that the energetic separation between the polariton and dark states is larger than a certain threshold of donor energetic disorder.

## Model System

Here, we outline the essentials part of the model, whereas the detailed expression of the full Hamiltonian is provided in the Supporting Information. An illustration of the model system is provided in Figure 1a, and the schematic energy diagram for the model system is depicted in Figure 1b. We consider a model photoinduced ET system that is coupled to a single-mode cavity at the Tavis–Cummings (TC) level of approximation.^1^ The total system Hamiltonian for the model is

1 $$Ĥ={Ĥ}_{m}+{Ĥ}_{p}+{Ĥ}_{mp}+{Ĥ}_{l}$$

where ${Ĥ}_{m}$ is the molecular Hamiltonian, ${Ĥ}_{p}$ is the photonic Hamiltonian, ${Ĥ}_{mp}$ describes the quantum light–matter interactions between molecules and photonic modes, and ${Ĥ}_{l}$ describes the continuous-wave (CW) laser driving of the hybrid system.

**Figure 1:**
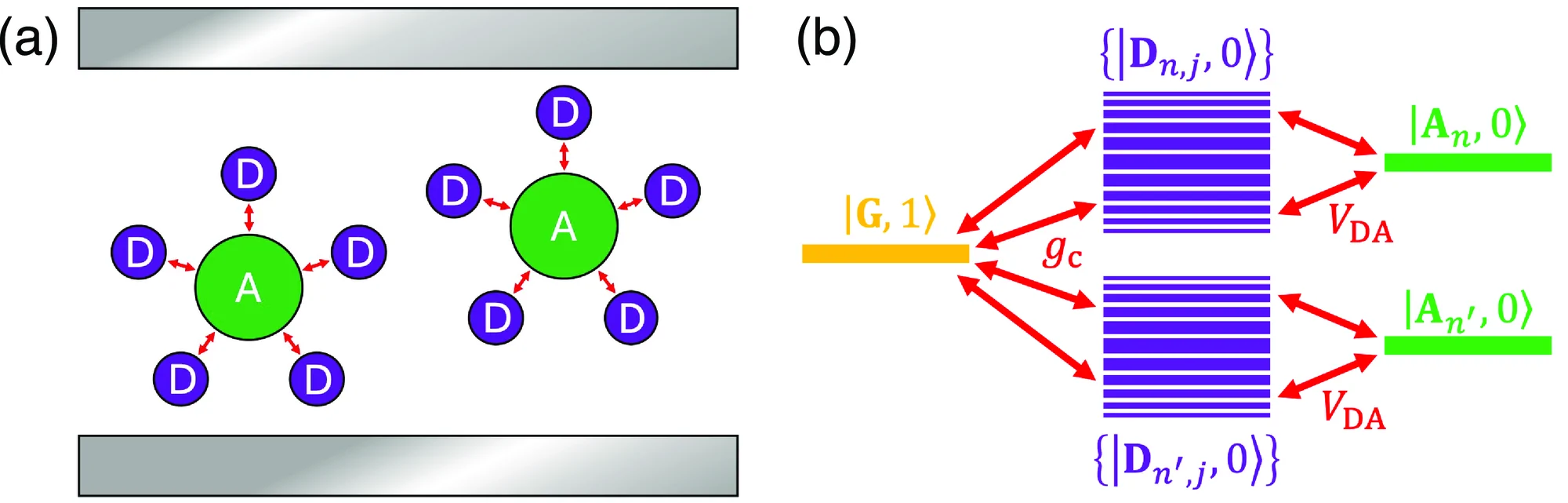
Super-reaction PMET model diagrams. (a) Illustration of multiple donor–acceptor clusters inside a Fabry–Pérot cavity. (b) Schematic energy levels of the system with couplings (red arrows) where all the donors couple to the cavity mode with individual coupling strength *g*_c_ and donors with label *n* locally couple to the *n*th acceptor with strength *V*_DA_.

The molecular Hamiltonian ${Ĥ}_{m}=\overset{N_{A}}{\underset{n=1}{\sum }}{Ĥ}_{n}$ describes a total of *N*_A_ super-reaction clusters, where *N*_A_ is the total number of acceptor states in the system. Each ${Ĥ}_{n}$ contains three types of electronic states, ground states $\left|G_{n,j}\right\rangle$ , donor states $\left|D_{n,j}\right\rangle$ , and an acceptor state $\left|A_{n}\right\rangle$ , with an ET reaction coordinate *R*_*n*,*j*_ (Marcus coordinate^52^) between each donor state and the acceptor state; *j* ∈ [1, *N*_SR_] indicates the label for the donor state.

The model Hamiltonian for a single super-reaction cluster is^35^

$$\begin{array}{ll} {Ĥ}_{n} & =\overset{N_{SR}}{\underset{j=1}{\sum }}[{\mathrm{T̂}}_{n,j}^{S}+\frac{1}{2}m_{S}{\omega_{S}}^{2}{R_{n,j}}^{2}\left|G_{n,j}\right\rangle \left\langle G_{n,j}\right|\\ & ,+\left(\Delta E_{D}+\frac{1}{2}m_{S}{\omega_{S}}^{2}{R_{n,j}}^{2}\right)\left|D_{n,j}\right\rangle \left\langle D_{n,j}\right|\\ & ,+\frac{1}{2}m_{S}{\omega_{S}}^{2}{\left(R_{n,j}-R_{A}^{0}\right)}^{2}\left|A_{n}\right\rangle \left\langle A_{n}\right|\\ & ,+V_{DA}\left(\left|D_{n,j}\right\rangle \langle A_{n}|+\left|A_{n}\right\rangle \langle D_{n,j}|\right)+{Ĥ}_{sb,n,j}]\\ & ,+\Delta E_{A}\left|A_{n}\right\rangle \left\langle A_{n}\right| \end{array}$$ 2

where *N*_SR_ is the number of donor states coupled to each acceptor state, *m*_S_ and ω_S_ are the mass and frequency, respectively, of the reaction coordinate with position *R*_*j*_, Δ*E*_D_ and Δ*E*_A_ are the energy shifts of the donor and acceptor states, respectively, with driving force Δ*G* = Δ*E*_A_ – Δ*E*_D_, $R_{A}^{0}$ is the minimum energy position of the acceptor states with reorganization energy $\lambda_{A}=\frac{1}{2}m_{S}{\omega_{S}}^{2}{R_{A}^{0}}^{2}$ , *V*_DA_ is the diabatic coupling between the donor and acceptor, ${\mathrm{T̂}}_{n,j}^{S}={P_{n,j}}^{2}/2m_{S}$ is the kinetic energy of the reaction coordinate with momentum *P*_*n*,*j*_, and ${Ĥ}_{sb,n,j}$ is the system-bath interaction. The donor states also have an associated reorganization energy λ_D_ which corresponds to the strength of the coupling between each donor state and its independent phonon bath (see the Supporting Information for details on the system-bath interaction).

Both donor and acceptor states are electronically excited states, and they are only coupled through local electronic couplings.

3 $$\left\langle D_{n,j}\left|{Ĥ}_{n}\right|A_{n}\right\rangle =V_{DA}$$

in the sense that, for $\left|A_{n}\right\rangle$ , there are a total of *N*_SR_ nearest neighbor donors coupled to it. The donor states have a bright transition dipole, described by

4 $$\left\langle D_{n,j}\left|{\mathrm{\mu ̂}}_{n,j}\right|G_{n,j}\right\rangle =\mu$$

whereas we assume the acceptor states are optically dark and have no transition dipole from the ground state. We make this assumption for the acceptor states to investigate how optically dark acceptor states can still become populated with photoexcitation to donor-polariton states and SR-PMET to the acceptor states.

The photonic Hamiltonian of a single quantized cavity mode is described as

5 $${Ĥ}_{p}=\hbar \omega_{c}\left({â}^{†}â+\frac{1}{2}\right)$$

where ω_c_ is the cavity frequency and â is the annihilation operator of the cavity mode. We further assume that all donor states $\left|D_{n,j}\right\rangle$ are optically coupled to the cavity with equal magnitude and dipole orientation, described by the following light–matter coupling

6 $${Ĥ}_{mp}=\overset{N_{A}}{\underset{n=1}{\sum }}\overset{N_{SR}}{\underset{j=1}{\sum }}g_{c}\left(â{\mathrm{\sigma ̂}}_{n,j}^{†}+{â}^{†}{\mathrm{\sigma ̂}}_{n,j}\right)$$

where *g*_c_ ∝ μ is the light–matter coupling strength between a single donor and the cavity mode, and ${\mathrm{\sigma ̂}}_{n,j}^{†}=\left|D_{n,j}\right\rangle \left\langle G_{n,j}\right|$ is the creation operator of the donor exciton state $\left|D_{n,j}\right\rangle$ . The total number of donors coupled to the cavity mode is

7 $$N_{D}=N_{SR}\cdot N_{A}$$

due to the nonlocal nature of the cavity field (eq 4), and *N*_SR_ = *N*_D_/*N*_A_ is the ratio between the number of donors and acceptors. To summarize, each donor is coupled to an acceptor molecule through a *chemically local* electronic coupling *V*_DA_, but all donors are coupled to the cavity through nonlocal light–matter interactions.

For the case λ_D_ = 0, where the donors have no coupling to their phonon bath, the donor state energies are identical for all nuclear positions **R** and the polariton states can be categorized as bright or dark states. The bright polariton states are the upper polariton (UP) and lower polariton (LP) states which are superpositions of singly excited donor states with 0 photons $\left|D_{n,j},0\right\rangle$ and ground states with 1 photon |G,1⟩ , defined as

8a $$\left|+\right\rangle =\sin\Phi \frac{1}{\sqrt{N_{D}}}\overset{N_{A}}{\underset{n=1}{\sum }}\overset{N_{SR}}{\underset{j=1}{\sum }}\left|D_{n,j},0\right\rangle +\cos\Phi \left|G,1\right\rangle$$

8b $$\left|-\right\rangle =\cos\Phi \frac{1}{\sqrt{N_{D}}}\overset{N_{A}}{\underset{n=1}{\sum }}\overset{N_{SR}}{\underset{j=1}{\sum }}\left|D_{n,j},0\right\rangle -\sin\Phi \left|G,1\right\rangle$$

where |+⟩ and |−⟩ are the UP and LP states, respectively, and the mixing angle Φ is

9 $$\Phi =\frac{1}{2}\;\tan^{-1}\left[\frac{2\sqrt{N_{D}}g_{c}}{\delta_{c}}\right]\in \left[0,\frac{\pi }{2}\right)$$

where the cavity detuning is δ_c_ = ℏω_c_ – Δ*E*_D_ – λ_D_. The energy difference between the UP and LP is known as the Rabi splitting, which can be expressed as

10 $$\Omega_{R}=\sqrt{4N_{D}{g_{c}}^{2}+{\delta_{c}}^{2}}$$

which only has direct dependence on *N*_D_ instead of *N*_A_ or *N*_SR_ separately. The remaining *N*_D_ – 1 eigenstates are the dark states $\left|D_{k}\right\rangle$ which are superpositions of only $\left\{\left|D_{n,j},0\right\rangle \right\}$ states. The energy of the dark states remains the same as the exciton site energy but with large density of states *N*_D_ – 1. Note that the dark states have no overlap with the collective “bright” states.

When the cavity is in resonance with the donor energy (Φ = π/4), the UP and LP states without disorder simplify to

11 $$\left|\pm \right\rangle =\frac{1}{\sqrt{2}}\left[\frac{1}{\sqrt{N_{D}}}\overset{N_{A}}{\underset{n=1}{\sum }}\overset{N_{SR}}{\underset{j=1}{\sum }}\left|D_{n,j},0\right\rangle \pm \left|G,1\right\rangle \right]$$

such that the UP and LP eigenstates are half donor character (equally distributed among all *N*_D_ donor molecules) and half photonic character. The Rabi splitting at resonance is $\Omega_{R}=2\sqrt{N_{D}}g_{c}$ . When the donor states are coupled to a bath (λ_D_ ≠ 0), the donor state energies may be disordered which causes the polariton eigenstates to no longer be perfectly bright or dark.^30,49,53,54^

## SR-PMET Rates

A key feature of the super-reaction model is the modification of the coupling strength between acceptor states and delocalized donor states.^33^ The UP state |+⟩ is delocalized across all donor states in the system and couples to the acceptor state $\left|A_{n},0\right\rangle$ as

12 $$\begin{array}{ll} V_{+A_{n}} & =\left\langle +\left|Ĥ\right|A_{n},0\right\rangle =\overset{N_{SR}}{\underset{j=1}{\sum }}\sin\Phi \cdot V_{DA}\cdot \frac{1}{\sqrt{N_{D}}}\\ & =\sin\Phi \cdot V_{DA}\cdot \sqrt{\frac{N_{SR}}{N_{A}}} \end{array}$$

where we have used eq 7, and the sum over *N*_SR_ locally coupled donors results in an overall coupling enhancement of $\sqrt{N_{SR}}$ . The transfer rates between these two states typically scale as $|V_{+A_{n}}{|}^{2}\propto N_{SR}/N_{A}$ , which is the super-reaction rate enhancement effect. Another effect of the super-reaction model is the increase in the reorganization energy between acceptor states and delocalized donor states. For a delocalized donor state such as the UP, the total reorganization energy relative to an acceptor state is

13 $$\lambda_{NA}\equiv N_{SR}\lambda_{A}$$

such that the reorganization energy scales with the number of donors coupled to each acceptor. This is due to the acceptor coupling to *N*_SR_ separate ET reaction coordinates which each contribute λ_A_ to the reorganization energy when the reaction is initiated from the minimum energy position of a delocalized donor state.

These modified coupling $V_{+A_{n}}$ and reorganization energy λ_NA_ values can be used to express the Marcus rate constant^30,52,55–58^ between states |+⟩ and $\left|A_{n},0\right\rangle$

14 $$k_{+A_{n}}=\frac{|V_{+A_{n}}{|}^{2}}{\hbar }\sqrt{\frac{\pi }{\lambda_{+A_{n}}k_{B}T}}\times \exp\left[-\frac{{\left(\Delta G_{+A_{n}}+\lambda_{+A_{n}}\right)}^{2}}{4\lambda_{+A_{n}}k_{B}T}\right]$$

where *k*_B_ is the Boltzmann constant, *T* is the temperature, and $\Delta G_{+A_{n}}$ is the effective driving force between |+⟩ and $\left|A_{n},0\right\rangle$ . The total SR-PMET rate constant *k*_+{**A**}_ in the Marcus regime between the UP and the acceptor state is

15 $$k_{+\left\{A\right\}}=\overset{N_{A}}{\underset{n=1}{\sum }}k_{+A_{n}}\propto N_{A}\cdot {\left(\sin\Phi \cdot V_{DA}\cdot \sqrt{\frac{N_{SR}}{N_{A}}}\right)}^{2}$$

where the factor *N*_A_ from the sum over the acceptor states cancels with the *N*_A_ in the denominator of the coupling. The total SR-PMET rate constant can thus be expressed as follows

16 $$k_{+\left\{A\right\}}=N_{SR}\cdot \sin^{2}\Phi \frac{{V_{DA}}^{2}}{\hbar }\sqrt{\frac{\pi }{\lambda_{NA}k_{B}T}}\times \exp\left[-\frac{{\left(\Delta G-\lambda_{D}-\frac{1}{2}\left(\delta_{c}+\Omega_{R}\right)+\lambda_{NA}\right)}^{2}}{4\lambda_{NA}k_{B}T}\right]$$

where $\Delta G_{+A_{n}}=\Delta G-\lambda_{D}-\frac{1}{2}\left(\delta_{c}+\Omega_{R}\right)$ is the effective driving force modified by the collective light–matter coupling and cavity detuning.^30^

For the case *N*_SR_ = *N*_D_/*N*_A_ = 1, this rate constant reduces to the “regular” PMET rate constant under the collective coupling regime (with *N* = *N*_A_) as derived in the previous work in ref 30, and under the single-molecule limit *N*_D_ = *N*_A_ = 1, it reduces back to the single-molecule PMET theory.^35^ Because the super-reaction enhancement of the coupling *N*_SR_ appears as a prefactor outside the exponential, it combines multiplicatively with the PMET enhancement due to the change of driving force with the collective Rabi splitting Ω_R_. Thus, a super-reaction ET system placed inside a cavity can experience enhancement from both the super-reaction and the collective Rabi splitting simultaneously.

The super-reaction enhancement described in eq 16 is sensitive to the coherence properties of the donor manifold. This enhancement is only observed for transfer originating from a bright donor state; any population residing in dark donor states prior to ET will reduce the observed super-reaction enhancement. Population transfer from bright states to dark states can occur due to the energetic disorder of the donor states, which is mediated by a phonon bath in the SR-PMET model in this work. To quantitatively understand the effects of this donor disorder on the super-reaction mechanism in the collective coupling regime, we explicitly simulate the dynamics of the SR-PMET model. In particular, we investigate how much donor disorder is needed to reduce or eliminate the super-reaction enhancement both inside and outside the cavity.

## Results and Discussion

In this Letter, we explore the population dynamics and rates of our SR-PMET model system in the collective coupling regime and compare them to the dynamics and rates outside the cavity. In all cases inside the cavity, we focus on cavities in resonance with the donor excited state (ℏω_c_ = Δ*E*_D_ + λ_D_ = 3 eV) in the Franck–Condon (FC) configuration and reactions initiated in, or coherently pumped to, the UP state. Simulations of the system outside the cavity instead have coherent pumping to the donor excited states. This regime of focus allows us to examine the extent to which the SR-PMET reaction rates can be significantly enhanced due to the super-reaction setup.

Figure 2a,b presents SR-PMET in the Marcus regime for varying numbers of donors *N*_SR_ coupled to each acceptor inside the cavity with a fixed λ_NA_ in order to isolate the effect of the enhanced coupling prefactor of the reaction rate in eq 16. The reaction inside the cavity is initiated as an FC excitation to the resonant UP state. Figure 2a shows the propagated and fitted acceptor state populations for varying *N*_SR_ ∈ [1, 5]. The system was propagated for 1 ps, and *k*_f_ and *k*_b_ were fitted based on eq 17 with the corresponding fitted populations from the right-hand side of eq 17 plotted as dashed lines. The dynamics show a nearly linear increase in acceptor population with increasing slope for larger *N*_SR_. The fitted populations show excellent agreement with the propagated populations, which demonstrates the rate-like behavior of the transfer from the UP to the acceptor even when multiple donor states are coupled to a given acceptor state along multiple reaction DOFs.

**Figure 2:**
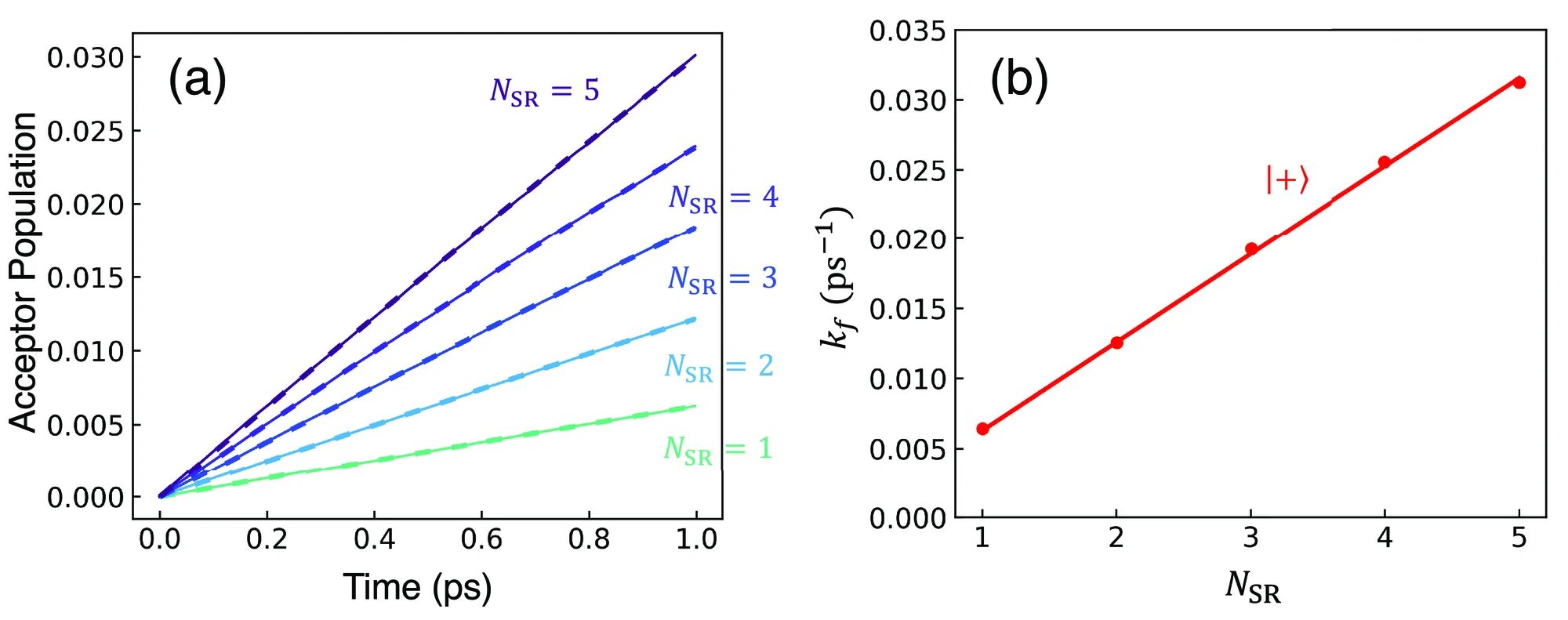
Super-reaction dynamics and rates inside the cavity with no donor disorder λ_D_ = 0. (a) Acceptor population dynamics when starting from the UP with *N*_SR_ ∈ [1, 5]. The solid lines are the propagated populations, and the dashed lines are the populations based on the fitted rate constants. (b) Forward rate constants when starting from the UP for *N*_SR_ ∈ [1, 5]. The solid line is the Marcus theory rate (eq 16), and the points are the fitted rates from the numerical propagation. Simulations were performed with $\sqrt{N_{D}}g_{c}=50$ meV, *N*_A_ = 100, λ_NA_ = 200 meV, *V*_DA_ = 1 meV, Δ*G* = 0, and *V*_l_ = 0.

Figure 2b shows the corresponding analytic Marcus forward rate constants (solid red line) and fitted forward rate constants (red dots) based on the explicit quantum dynamics propagation. The Marcus rate constants based on eq 16 show a perfect linear trend with *N*_SR_ since the total acceptor reorganization energy λ_NA_ has been fixed. The Marcus and fitted rate constants show excellent agreement for the entire parameter range of *N*_SR_ ∈ [1, 5]. This validates both the MFE simulation for the super-reaction model in the Marcus regime and the form of the analytic rate constant in eq 16 which describes the combined rate enhancement effect of both the super-reaction (due to *N*_SR_ in the prefactor) and the collective light–matter coupling (due to Ω_R_ in the driving force) as the product of the individual enhancements. The corresponding rate constant from the donor bright state to the acceptor for a super-reaction system outside the cavity with no disorder would show a similar linear dependence as the UP rate constant in Figure 2b but with a different overall magnitude due to the lack of driving force modification that appears inside the cavity for the UP state.

Figure 3 presents the SR-PMET fitted rate constant dependence on *N*_SR_ in the presence of donor disorder (λ_D_ = 20 meV) both inside and outside the cavity. The simulations were initiated in the global ground state and driven to the excited states through a continuous-wave (CW) laser coupling (see Supporting Information) with coupling strength *V*_l_ = 10 meV over 10 ps of propagation. The CW laser was tuned to the UP energy inside the cavity (red) and the matter energy Δ*E*_D_ + λ_D_ outside the cavity (black). This explicit laser driving allows for a more realistic excitation of the system compared to initializing the system in a perfect bright state. The fitted rate constants inside and outside the cavity are similar for *N*_SR_ = 1 due to the competing effects of the driving force enhancement due to Ω_R_ and the coupling strength suppression due to sin^2^Φ in eq 16. As *N*_SR_ increases, however, a clear difference emerges in the trends inside and outside the cavity. The rate constants inside the cavity increase linearly with *N*_SR_, similar to those in Figure 2b, due to the super-reaction enhancement effect of *N*_SR_. This is in spite of the donor disorder of λ_D_ = 20 meV, which demonstrates that the collective cavity coupling can protect the coherent super-reaction enhancement from moderate-strength disorder. The rate constants outside the cavity instead show no significant increase (or decrease) in rate with increasing *N*_SR_. This demonstrates that a system outside the cavity does not experience any super-reaction enhancement when moderate donor disorder is present, at least in this ET model system. The lack of any change of rate outside the cavity for increasing *N*_SR_ can be understood in the bright/dark representation of the donor states: the disorder causes donor population to distribute equally among the bright and dark states such that the bright donor states have a population of 1/*N*_SR_ before transfer, which cancels out the super-reaction enhancement of the bright state. In contrast, the energetic separation between the UP and dark states inside the cavity prevents the population transfer from the bright UP to the dark states.

**Figure 3:**
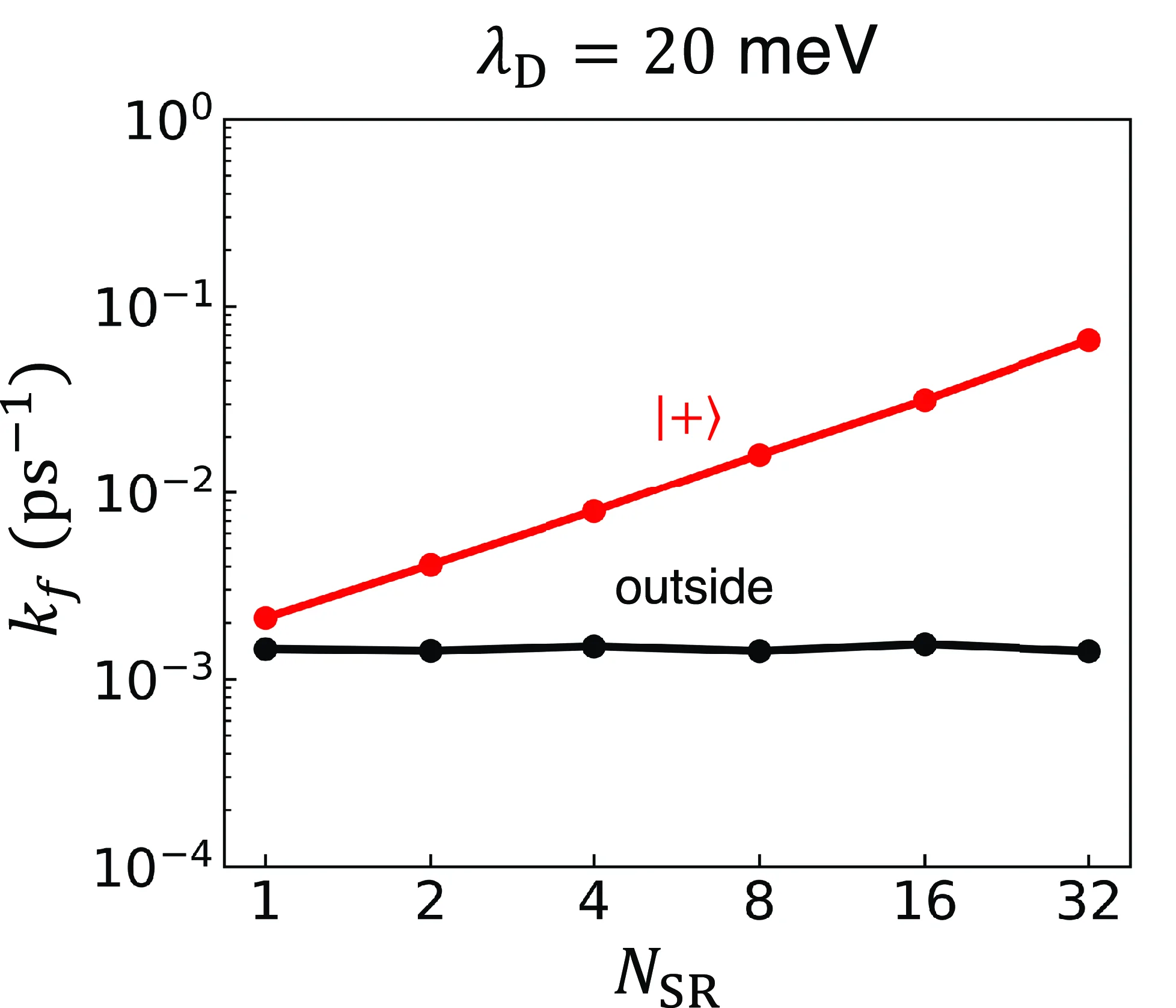
Super-reaction fitted rate constants with donor disorder λ_D_ = 20 meV as a function of *N*_SR_ ∈ [1, 32]. The simulations were initiated from the global ground state |G,0⟩ , and CW laser driving was tuned to the UP energy with light–matter coupling $\sqrt{N_{D}}g_{c}=150$ meV inside the cavity (red) and the matter energy Δ*E*_D_ + λ_D_ outside the cavity (black). Simulations were performed with *N*_A_ = 100, λ_NA_ = 200 meV, *V*_DA_ = 0.5 meV, Δ*G* – λ_D_ = 0, and *V*_l_ = 10 meV. The total number of donors *N*_D_ = *N*_SR_·*N*_A_ is up to 3200.

Figure 4 presents the SR-PMET fitted rate constant dependence on λ_D_ with a fixed donor-to-acceptor ratio of *N*_SR_ = 32 both inside and outside the cavity. The CW laser excitation and propagation time are the same as in Figure 3. Two collective coupling strengths, $\sqrt{N_{D}}g_{c}=300$ meV (red) and $\sqrt{N_{D}}g_{c}=100$ meV (blue), were tested inside the cavity to demonstrate how the magnitude of the collective coupling affects the effect, or lack thereof, of donor disorder on the super-reaction enhancement. The rate constants for the $\sqrt{N_{D}}g_{c}=300$ meV simulations show little dependence on λ_D_, indicating that donor disorder λ_D_ ≤ 50 meV is not large enough to impact the super-reaction enhancement of the UP to acceptor transfer. On the other hand, the rate constants for the $\sqrt{N_{D}}g_{c}=100$ meV simulations show a significant decrease of rate for larger values of λ_D_ with the significant decrease in rate appearing around λ_D_ ≈ 25 meV. This donor reorganization energy corresponds to an energetic broadening of around^59^ $\sigma_{D}=\sqrt{2\lambda_{D}k_{B}T}\approx 36$ meV which is slight larger than 1/3 of $\sqrt{N_{D}}g_{c}$ . This is consistent with the criteria that an energetic broadening of $\sigma_{D}\geq \sqrt{N_{D}}g_{c}/3$ will cause significant population transfer from bright polariton states to dark states inside cavities.^30,60^ As more population transfers to dark states for larger values of λ_D_, the rate constants for $\sqrt{N_{D}}g_{c}=100$ meV decrease due to the slower ET rates of the dark states, which do not experience super-reaction enhancement. This demonstrates that, in order to observe super-reaction enhancement inside the cavity, the polariton and dark states must be sufficiently energetically separated relative to the donor disorder width to prevent population transfer to dark states.

**Figure 4:**
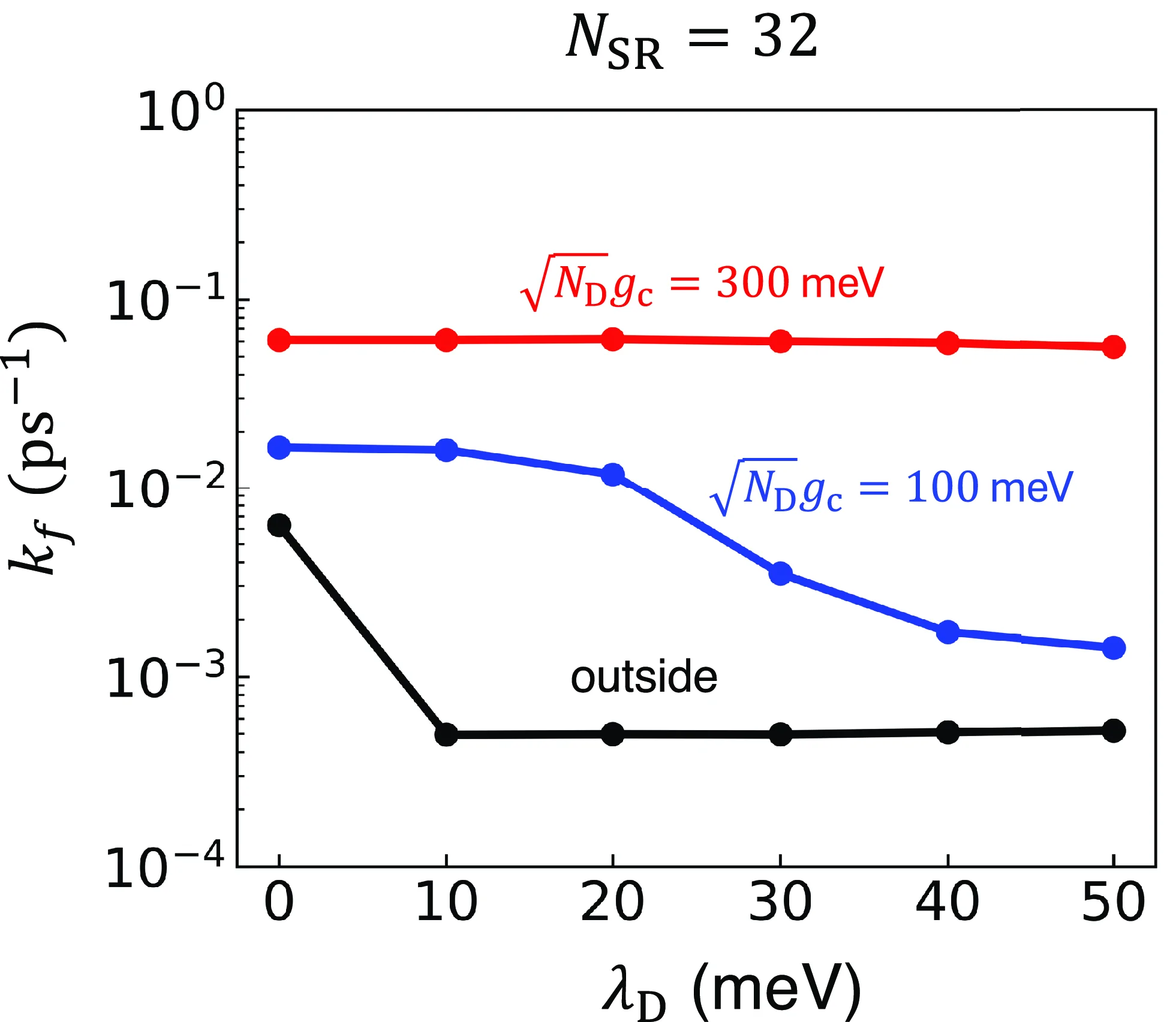
Super-reaction fitted rate constants with *N*_SR_ = 32 donors per acceptor state as a function of donor disorder λ_D_ ∈ [0, 50] meV. The simulations were initiated from the global ground state |G,0⟩ , and CW laser driving was tuned to the UP energy with light–matter coupling $\sqrt{N_{D}}g_{c}=300$ meV (red) and $\sqrt{N_{D}}g_{c}=100$ meV (blue) inside the cavity and the matter energy Δ*E*_D_ + λ_D_ outside the cavity (black). Simulations were performed with *N*_A_ = 100, λ_NA_ = 300 meV, *V*_DA_ = 0.5 meV, Δ*G* – λ_D_ = 0, and *V*_l_ = 10 meV.

The rate constants outside the cavity in Figure 4 show a more severe dependence on donor disorder with λ_D_. The rate constant outside the cavity for λ_D_ = 10 meV is 10 times smaller than for λ_D_ = 0, indicating that nearly all of the super-reaction enhancement present in the disorderless case is eliminated even for relatively small donor disorder. This is unsurprising, given that there is no mechanism outside the cavity to prevent transfer from bright to dark donor states in the presence of any disorder. Additionally, there is a small increase in rate outside the cavity from λ_D_ = 10 meV to λ_D_ = 50 meV. This increase can be explained by considering the effect of donor energy disorder on the driving force Δ*G*. Donor states that happen to have a higher energy will have a more favorable Δ*G* and faster transfer to acceptor states than donor states with lower energies for these ET parameters. Because the rate dependence on Δ*G* in eq 16 is nonlinear, a symmetric distribution of dark donor state energies around their mean will lead to an asymmetric distribution of rate constants such that the rate constant averaged over all the dark donor states will be slightly larger for larger donor disorder λ_D_. This effect also influences the rate constants of $\sqrt{N_{D}}g_{c}=100$ meV simulations inside the cavity for λ_D_ ≥ 30 meV since the dark donor states are populated in this case. Regardless, this effect is negligible compared to the super-reaction enhancement of the bright states in this model.

In this Letter, we examined the effects of the super-reaction mechanism on PMET in the large *N*_A_ regime. The prefactor of the Marcus rate constant from the bright polariton states to the acceptor states, which contains the super-reaction enhancement that scales linearly with *N*_SR_, is independent of *N*_A_ due to the sum over the contributions from all acceptor states. This causes super-reaction enhancement in any parameter regime, provided that the bright polariton states are the donor states. Further, this prefactor enhancement combines multiplicatively with the driving force modification due to collective light–matter coupling in PMET, allowing for both enhancements to be present without competition.

We performed mixed quantum-classical simulations of the system dynamics to understand the impacts of donor disorder on the super-reaction mechanism. In the limit of no donor disorder (λ_D_ = 0), the fitted rate constants from the simulations match those of the analytic Marcus rate constant with linear *N*_SR_ scaling, validating the ability of the simulation to capture the super-reaction mechanism in PMET for large *N*_A_ = 100. When moderate donor disorder is added, the fitted rate constants from the UP maintain their linear *N*_SR_ scaling, while the outside cavity rates show no enhancement with *N*_SR_ due to the inability to protect donor coherence from disorder. This explains why a typical ET reaction outside the cavity is not expected to experience a super-reaction enhancement even when multiple donors are locally coupled to individual acceptors.

To quantitatively understand how much donor disorder can be present while preserving the super-reaction enhancement, we performed a simulation scan over λ_D_. The results revealed that the bright polariton states maintain their super-reaction enhancement as long as the donor disorder width is less than a third of the collective coupling strength at resonance, while the enhancement is lost outside the cavity even for small donor disorder widths. This criterion is similar to the criterion for observing driving force modification in PMET^30^ and other polariton phenomena^60^ with the common denominator that the system must mostly remain in the bright, delocalized polariton state in order to experience the unique effects of this state. This result offers a design principle where, through either creating large collective light–matter coupling or positively detuning the UP away from the dark states, a molecular ET system with multiple donors coupled to each acceptor can experience super-reaction enhancement even with phonon-induced disorder.

## Theoretical Approaches

The reduced density matrix dynamics ρ̂(t) of the molecule-cavity SR-PMET system in this work were propagated at the wave function level using the mean-field Ehrenfest (MFE) approach.^61^ MFE is a mixed quantum-classical dynamics method that propagates a quantum subsystem (electrons and photons) alongside a classical subsystem (nuclei), which feels a mean-field nuclear force based on the quantum state.^62–64^ The reaction coordinate and donor phonon baths, described by ${Ĥ}_{sb,n,j}$ , were treated implicitly using a Langevin dynamics approach, which is equivalent to the reduced dynamics of the harmonic ET coordinate under the influence of an Ohmic bath in the Markovian regime.^35,65–67^ Further details of the simulations can be found in the Supporting Information.

To compute rate constants based on explicit simulations, we fit the simulated population dynamics of the acceptor states $\rho_{\left\{A\right\}}\left(t\right)\equiv \underset{n}{\sum }\left\langle A_{n}\right|\mathrm{\rho ̂}\left(t\right)\left|A_{n}\right\rangle$ to the following equation

17 $$\rho_{\left\{A\right\}}\left(t\right)=\frac{k_{f}}{k_{f}+k_{b}}\left(1-\exp\left[-\left(k_{f}+k_{b}\right)t\right]\right)$$

where *k*_f_ and *k*_b_ are the fitted effective forward and backward rate constants, respectively, between the donor and acceptor manifolds.

## Supplementary Material



## Notes

Model Hamiltonian Details and Quantum Dynamics Simulation Details (PDF)
