# Supplementary material for: Super-Reaction Enhancement of Polariton-Mediated Electron Transfer
Source: J Phys Chem Lett. 2026 Oct 27;17(44):12638–45. doi: 10.1021/acs.jpclett.6c03034 (PMC13746818; doi:10.1021/acs.jpclett.6c03034)
Supplement: Supplementary file 1 [file jz6c03034_si_001.pdf]

Supporting Information: Super-Reaction Enhancement of Polariton Mediated Electron Transfer

Eric R. Koessler,*,† Todd D. Krauss,†,‡,¶ and Pengfei Huo*,†,‡,¶

†*Department of Chemistry, University of Rochester, Rochester, NY 14627, U.S.A.*

‡*The Institute of Optics, Hajim School of Engineering, University of Rochester, Rochester, NY 14627, U.S.A.*

¶*Center for Coherence and Quantum Science, University of Rochester, Rochester, New York 14627, USA*

E-mail: ekoessle@ur.rochester.edu; pengfei.huo@rochester.edu

Model Hamiltonian

The system-bath interaction $\hat{H}_{\mathrm{sb},n,j}$ can be expressed as

$$\hat{H}_{\mathrm{sb},n,j} = \hat{H}^{\mathrm{CL}}_{\mathrm{sb},n,j} + \hat{H}^{\mathrm{D}}_{\mathrm{sb},n,j}. \tag{S1}$$

The term $\hat{H}^{\mathrm{CL}}_{\mathrm{sb},n,j}$ is the Caldeira-Leggett system-bath interaction[1] of the reaction coordinate with a phonon bath

$$\hat{H}^{\mathrm{CL}}_{\mathrm{sb},n,j} = \sum_u \left[\frac{P^2_{u,n,j}}{2m_u} + \frac{1}{2}m_u\omega_u^2\left(R_{u,n,j} - \frac{c_u}{m_u\omega_u^2}R_{n,j}\right)^2\right], \tag{S2}$$

where $R_{u,n,j}$ and $P_{u,n,j}$ are the position and momentum, respectively, of bath mode u, and ω_u and c_u are the frequency and coupling strength, respectively, of bath mode u that are described with the Ohmic spectral density $J_{\mathrm{CL}}(\omega) = \zeta\omega e^{-\omega/\omega_0}$. The friction parameter ζ determines the system-bath coupling strength and the high frequency cut-off is $\omega_0 \gg \omega_{\mathrm{S}}$. The term $\hat{H}^{\mathrm{D}}_{\mathrm{sb},n,j}$ is the Holstein

system-bath interaction of the donor states with a phonon bath

$$\hat{H}^{\mathrm{D}}_{\mathrm{sb},n,j} = \sum_{v} \left[\frac{P^2_{v,n,j}}{2m_v} + \frac{1}{2} m_v \omega_v^2 \left(R_{v,n,j} - \sqrt{\frac{2\hbar S_v}{m_v \omega_v}} |\mathrm{D}_{n,j}\rangle\langle \mathrm{D}_{n,j}| \right)^2 \right], \tag{S3}$$

where $R_{\nu,n,j}$ and $P_{\nu,n,j}$ are the position and momentum, respectively, of bath mode ν, and ω_ν and S_ν are the frequency and Huang-Rhys factor, respectively, of bath mode ν. The donor bath kinetic energy is $\hat{T}^{\mathrm{D}}_{n,j} = \sum_\nu P^2_{\nu,n,j}/2m_\nu$ and the donor reorganization energy is $\lambda_{\mathrm{D}} = \sum_\nu \hbar S_\nu \omega_\nu$. For simplicity, we model the spectral density of the donor bath as a Brownian oscillator $J_{\mathrm{D}}(\omega) = 2\lambda_{\mathrm{D}}\gamma\omega_{\mathrm{S}}^2\omega/\pi((\omega_{\mathrm{S}}^2-\omega^2)^2+\gamma^2\omega^2)$ with the same parameters ω_{S} and $\gamma = \zeta/m_{\mathrm{S}}$ as the effective Brownian oscillator spectral density of the reaction coordinate bath[2] aside from the donor reorganization energy λ_{D}. During the MFE simulations of the system, we use a Langevin bath coupled to harmonic oscillators[3] for both the reaction coordinates with Caldeira-Leggett interaction (acceptor bath) as well as the donor-bath dynamics, with the only distinguishing parameters being the reorganization energies λ_{D} and λ_{A}.

We focus on the single excitation subspace.[4,5] This includes the collective ground state $|\mathbf{G}\rangle$ and singly excited states $|\mathbf{D}_{n,j}\rangle$ and $|\mathbf{A}_n\rangle$ (where $n \in [1, N_{\mathrm{A}}]$ labels the super-reaction clusters), defined as

$$|\mathrm{G}_n\rangle \equiv |\mathrm{G}_{n,1}\rangle \otimes ... \otimes |\mathrm{G}_{n,N_{\mathrm{SR}}}\rangle, \tag{S4a}$$

$$|\mathrm{D}'_{n,j}\rangle \equiv |\mathrm{G}_{n,1}\rangle \otimes ... \otimes |\mathrm{D}_{n,j}\rangle \otimes ... \otimes |\mathrm{G}_{n,N_{\mathrm{SR}}}\rangle, \tag{S4b}$$

$$|\mathbf{G}\rangle \equiv |\mathrm{G}_1\rangle \otimes ... \otimes |\mathrm{G}_{N_{\mathrm{A}}}\rangle, \tag{S4c}$$

$$|\mathbf{D}_{n,j}\rangle \equiv |\mathrm{G}_1\rangle \otimes ... \otimes |\mathrm{D}'_{n,j}\rangle \otimes ... \otimes |\mathrm{G}_{N_{\mathrm{A}}}\rangle, \tag{S4d}$$

$$|\mathbf{A}_n\rangle \equiv |\mathrm{G}_1\rangle \otimes ... \otimes |\mathrm{A}_n\rangle \otimes ... \otimes |\mathrm{G}_{N_{\mathrm{A}}}\rangle. \tag{S4e}$$

The cavity mode in the single excitation subspace is treated as a two-level system with states $|0\rangle$, which is the vacuum 0-photon Fock state whose energy is set to 0 for convenience, and $|1\rangle$, which is the 1-photon Fock state with energy $\hbar\omega_c$. In the combined molecule-cavity Hilbert space, which is restricted to zero and single-excitation states, we label the states in a condensed fashion (e.g., $|\mathbf{G}\rangle \otimes |1\rangle \equiv |\mathbf{G}, 1\rangle$).

The CW laser Hamiltonian $\hat{H}_\mathrm{l}$ describes a continuous driving between the cavity states of the system[6] and is expressed as

$$\hat{H}_\mathrm{l}(t) = V_\mathrm{l}\left(\hat{a}e^{i\omega_\mathrm{l}t} + \hat{a}^\dagger e^{-i\omega_\mathrm{l}t}\right), \tag{S5}$$

where V_l is the laser coupling strength and ω_l is the frequency of the laser. This laser model assumes the external laser is directly coupling to the cavity mode through a partially-reflective mirror or other cavity structure.[7] For simulations outside the cavity, the corresponding CW laser Hamiltonian $\hat{H}_\mathrm{l}^\mathrm{out}$ is

$$\hat{H}_\mathrm{l}^\mathrm{out}(t) = V_\mathrm{l}\left(\hat{B}e^{i\omega_\mathrm{l}t} + \hat{B}^\dagger e^{-i\omega_\mathrm{l}t}\right), \tag{S6}$$

where $\hat{B}^\dagger = \frac{1}{\sqrt{N_\mathrm{D}}}\sum_{n=1}^{N_\mathrm{A}}\sum_{j=1}^{N_\mathrm{SR}}|\mathbf{D}_{n,j},0\rangle\langle\mathbf{G}|$ is the bright donor creation operator which models the laser field directly coupling to the donors outside the cavity through their transition dipoles.

We further define the polaritonic Hamiltonian as

$$\begin{aligned}\hat{H}_\mathrm{pl} =& \sum_{n=1}^{N_\mathrm{A}}\hat{\mathcal{P}}_n\Big(\hat{H}_n - \sum_{j=1}^{N_\mathrm{SR}}\big(\hat{T}_{n,j}^\mathrm{S} + \hat{T}_{n,j}^\mathrm{D} + \hat{H}_{\mathrm{sb},n,j}^\mathrm{CL}\big)\Big)\hat{\mathcal{P}}_n \\ &+ \hat{H}_\mathrm{p} + \hat{H}_\mathrm{mp},\end{aligned} \tag{S7}$$

where $\hat{\mathcal{P}}_n = \sum_j |\mathrm{G}_{n,j}\rangle\langle\mathrm{G}_{n,j}| + |\mathrm{D}_{n,j}\rangle\langle\mathrm{D}_{n,j}|$ is the projection operator that projects onto the ground and donor molecular states which excludes the acceptor states from $\hat{H}_\mathrm{pl}$.

The eigenstates of $\hat{H}_\mathrm{pl}$ are the so-called polariton states of the system. For a given nuclear configuration $\mathbf{R} \equiv \{R_{n,j}, R_{\nu,n,j}\}$, these polariton states $|\psi_a(\mathbf{R})\rangle$ and their corresponding eigenenergies $E_a(\mathbf{R})$ can be determined by the following eigenvalue equation

$$\hat{H}_\mathrm{pl}(\mathbf{R})|\psi_a(\mathbf{R})\rangle = E_a(\mathbf{R})|\psi_a(\mathbf{R})\rangle, \tag{S8}$$

where a labels the eigenstates. The polariton states with a single excitation can be expanded in the diabatic-Fock basis as

$$|\psi_a(\mathbf{R})\rangle = c_{a,0}|\mathbf{G},1\rangle + \sum_{n=1}^{N_\mathrm{A}}\sum_{j=1}^{N_\mathrm{SR}} c_{a,n,j}|\mathbf{D}_{n,j},0\rangle, \tag{S9}$$

where $c_{a,0}$ is the eigenstate expansion coefficient of the ath polariton state for $|\mathbf{G},1\rangle$ and $c_{a,n,j}$ is the eigenstate expansion coefficient of the ath polariton state for $|\mathbf{D}_{n,j},0\rangle$.

For the case $\lambda_{\mathrm{D}} = 0$, the bright polariton states are given in Eq. 8a. The remaining $N_{\mathrm{D}} - 1$ eigenstates are the dark states $|\mathcal{D}_k\rangle$ (not to be confused with the donor exciton states $|\mathbf{D}_{n,j},0\rangle$) which are superpositions of only $\{|\mathbf{D}_{n,j},0\rangle\}$ states that can be expressed as follows[4,8,9]

$$|\mathcal{D}_k\rangle = \frac{1}{\sqrt{N_{\mathrm{D}}}} \sum_{n=1}^{N_{\mathrm{A}}} \sum_{j=1}^{N_{\mathrm{SR}}} \exp\left(-2\pi i \frac{((n-1)N_{\mathrm{SR}} + j)k}{N_{\mathrm{D}}}\right) |\mathbf{D}_{n,j},0\rangle, \tag{S10}$$

where $k \in \{1, \cdots, N_{\mathrm{D}} - 1\}$ labels the dark states. Note that the dark states have no overlap with the collective "bright" states, and they do not participate in the interaction with the cavity mode mediated by $\hat{H}_{\mathrm{mp}}$ since $\sum_{n=1}^{N_{\mathrm{A}}} \sum_{j=1}^{N_{\mathrm{SR}}} c_{k,n,j} = 0$, which renders them optically dark (i.e. having no transition dipole from the ground state $|\mathbf{G}\rangle$).

Simulation Details

The mean-field Ehrenfest (MFE) method with Langevin dynamics was used to propagate the populations of the ground and excited quantum states of the system under the influence of the classical nuclear baths. Each acceptor state was coupled to N_{SR} Marcus reaction coordinates (harmonic oscillators) with Langevin damping and each donor state was coupled to its own separate nuclear harmonic oscillator with Langevin damping. The CW laser used to drive the excited state populations was propagated using the analytic solution to CW driving dynamics between two quantum states.[3] The cost of the Hamiltonian propagation was reduced by manually encoding the result of the Hamiltonian matrix multiplying the wavefunction vector such that the matrix-vector multiplication scaled linearly with N_{states} instead of N_{states}^2.[10]

Across all simulations, the bath parameters used are $\hbar\omega_{\mathrm{S}} = 9.54$ meV, $\hbar\gamma = 64.1$ meV, and $T = 300$ K for consistency with previous works on ET.[3,11,12] The initial positions and momenta of the harmonic oscillators for the donor and acceptor baths are sampled from a Wigner distribution

of the nuclear density operator $\hat{\rho}_{\mathbf{R}}$ as follows

$$(\hat{\rho}_{\mathbf{R}})^W \propto \Pi_{n,j} \exp\Big[-\tanh\Big(\frac{\omega_\mathrm{S}}{2k_\mathrm{B}T}\Big)\cdot\Big(m_\mathrm{S}\omega_\mathrm{S}R_{n,j}^2+\frac{P_{n,j}^2}{m_\mathrm{S}\omega_\mathrm{S}}\Big)\Big]. \tag{S11}$$

The number of trajectories was $N_\mathrm{traj} = 64,000/N_\mathrm{D}$ due to the self-averaging of the sum of the donor states and sum of the acceptor states when multiple molecules are coupled to the cavity. The nuclear time step was $\mathrm{dt_N} = 0.125$ fs and the electronic time step was $\mathrm{dt_E} = \mathrm{dt_N}/4$.

References

(1) Caldeira, A. O.; Leggett, A. J. Influence of Dissipation on Quantum Tunneling in Macroscopic Systems. *Phys. Rev. Lett.* **1981**, *46*, 211–214.

(2) Garg, A.; Onuchic, J. N.; Ambegaokar, V. Effect of friction on electron transfer in biomolecules. *The Journal of Chemical Physics* **1985**, *83*, 4491–4503.

(3) Koessler, E. R.; Mandal, A.; Musser, A. J.; Krauss, T. D.; Huo, P. Polariton mediated electron transfer under the collective molecule–cavity coupling regime. *Chem. Sci.* **2025**, *16*, 11644–11658.

(4) Mandal, A.; Taylor, M. A.; Weight, B. M.; Koessler, E. R.; Li, X.; Huo, P. Theoretical advances in polariton chemistry and molecular cavity quantum electrodynamics. *Chemical Reviews* **2023**, *123*, 9786–9879.

(5) Mauro, L.; Caicedo, K.; Jonusauskas, G.; Avriller, R. Charge-Transfer Chemical Reactions in Nanofluidic Fabry-Pérot Cavities. *Phys. Rev. B* **2021**, *103*, 165412.

(6) Qiu, L.; Mandal, A.; Morshed, O.; Meidenbauer, M. T.; Girten, W.; Huo, P.; Vamivakas, A. N.; Krauss, T. D. Molecular Polaritons Generated from Strong Coupling between CdSe Nanoplatelets and a Dielectric Optical Cavity. *J. Phys. Chem. Lett.* **2021**, *12*, 5030–5038.

(7) Herrera, F.; Spano, F. C. Absorption and Photoluminescence in Organic Cavity QED. *Phys. Rev. A* **2017**, *95*, 053867.

(8) Li, T. E.; Cui, B.; Subotnik, J. E.; Nitzan, A. Molecular Polaritonics: Chemical Dynamics Under Strong Light–Matter Coupling. *Annual Review of Physical Chemistry* **2022**, *73*, 43–71.

(9) Lai, Y.; Ying, W.; Huo, P. Non-equilibrium rate theory for polariton relaxation dynamics. *The Journal of Chemical Physics* **2024**, *161*, 104109.

(10) Mondal, M. E.; Vamivakas, A. N.; Cundiff, S. T.; Krauss, T. D.; Huo, P. Polariton spectra under the collective coupling regime. I. Efficient simulation of linear spectra and quantum dynamics. *The Journal of Chemical Physics* **2025**, *162*, 014114.

(11) Mandal, A.; Krauss, T. D.; Huo, P. Polariton-Mediated Electron Transfer via Cavity Quantum Electrodynamics. *J. Phys. Chem. B* **2020**, *124*, 6321–6340.

(12) Schwerdtfeger, C. A.; Soudackov, A. V.; Hammes-Schiffer, S. Nonadiabatic dynamics of electron transfer in solution: Explicit and implicit solvent treatments that include multiple relaxation time scales. *The Journal of Chemical Physics* **2014**, *140*, 034113.
